# Supplementary material for: Development of flipped classroom module fcm for music theory instruction: An innovative approach to music education
Source: PLoS One. 2025 Nov 21;20(11):e0337590. doi: 10.1371/journal.pone.0337590 (PMC12637912; doi:10.1371/journal.pone.0337590)

**Pre-test/Post-test instrument**

**Name :__________________________**

**Student NO :__________________________**

**_________________________________________________________**

**Problem-based flipped classroom**

**FCM module pre-test/post-test assessment**

**============================================================**

**Music Theory Course**

**Semester: 01**

**Student Cognitive Abilities and Music Element Skills**

**Exam duration: 1 hour 30 minutes**

**============================================================**

**Do not open this exam paper until you receive notification.**

**General instructions**

**1. Candidates should read the questions carefully and answer all questions.**

**2. Answer all questions on the exam paper.**

**3. Do not take the exam paper out of the exam room.**

**A: Test Paper: Student Cognitive Abilities (Pre-test)**

**Name: ______________________ Date: _________________________**

**Instructions: Single Choice Question（25）**

**1.Arranging the tones in a musical system according to certain pitch relationships and order is called ( ).**

- 1. A. Pitch
  2. B. Tone row
  3. C. Scale
  4. D. Tone group

**2.**
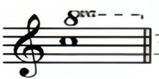
 **Which group do the notes in the staff belong to ( ) .**

- 1. A. Small character group c
  2. B. Small character group 1 c
  3. C. Small character group 2 c
  4. D. Small character group 3 c

**3.
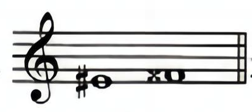
** **The relationship between these two sounds is ( ).**

- 1. A. Natural whole tone
  2. B. Altered whole tone
  3. C. Natural half tone
  4. D. Altered half tone

**4.
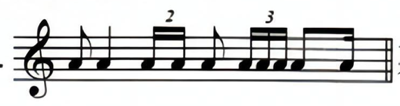
** **Its total time value is equal to ( ).**


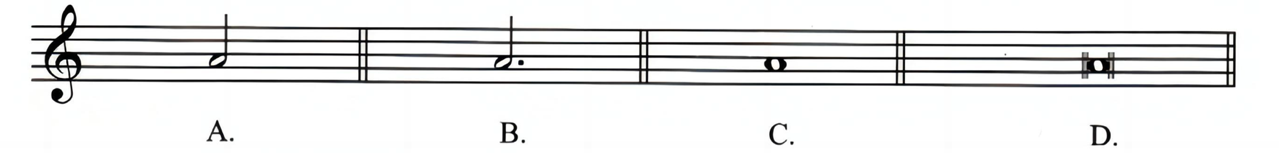


**5.Which of the following four musical terms is an expression term ( ).**

- 1. A. Lento
  2. B. ritenuto
  3. C. assai
  4. D. cantabile

**6.The interval with a number of tones equal to 3 is ( ).**

- 1. A. Double fourth
  2. B. diminished fifth
  3. C. perfect fifth
  4. D. diminished sixth

**7.The transposition of a major sixth interval is ( ).**

- 1. A. Altered interval
  2. B. Perfect interval
  3. C. Imperfect interval
  4. D. Dissonant interval

**8.Which of the following four intervals is not a natural interval ( ).**


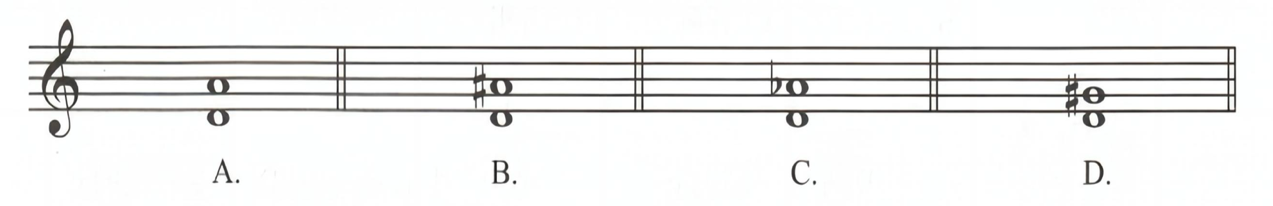


**9.Which of the following four chords is a consonant chord ( ).**


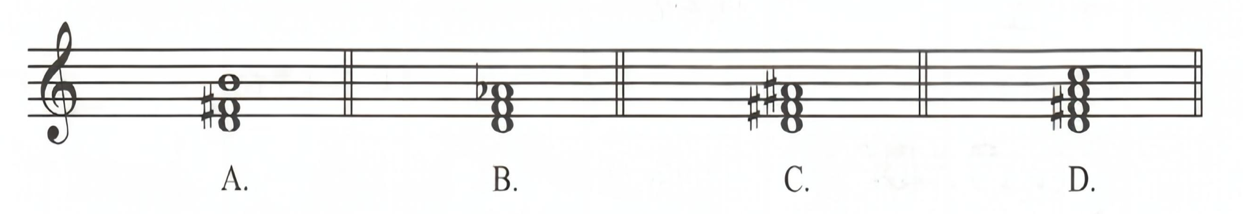


**10.** **Which of the following four chords is a half-diminished seventh chord ( ).**

**
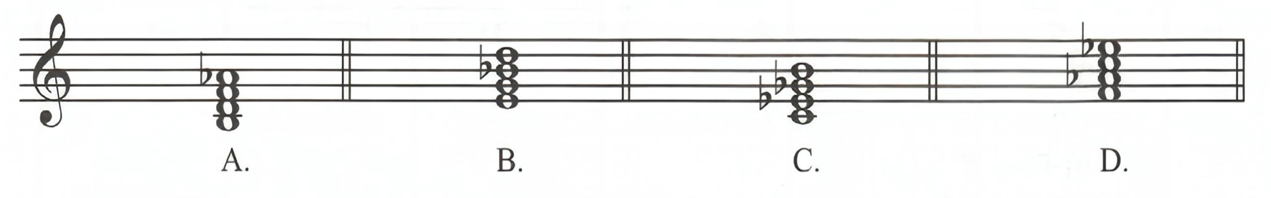
**

**11.The following are not
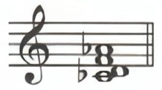
 equal chords ( ).**


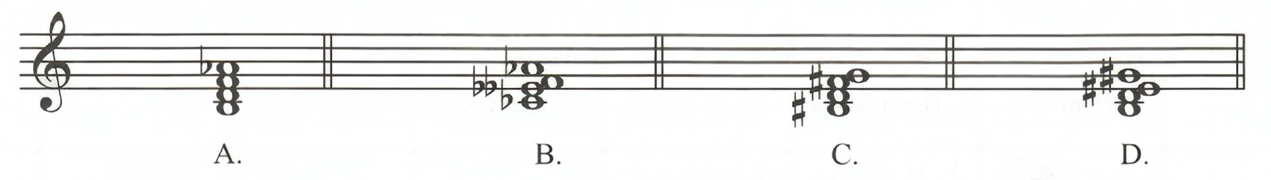


**12.In all modes, the augmented second interval exists in ( ).**

- 1. A. Natural major and minor scales
  2. B. Harmonic major and minor scales
  3. C. Melodic major and minor scales
  4. D. Seven-tone classical music

**13.Which of the following chords does not belong to the major triad of D major ( ).**
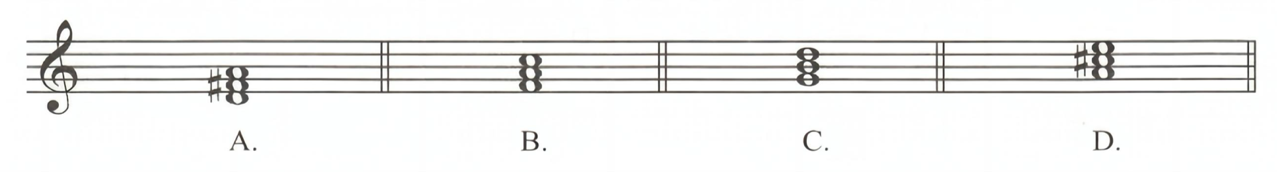


**14.See the following examples. In numbers ① to ④, which ones are in group f3( ) .**


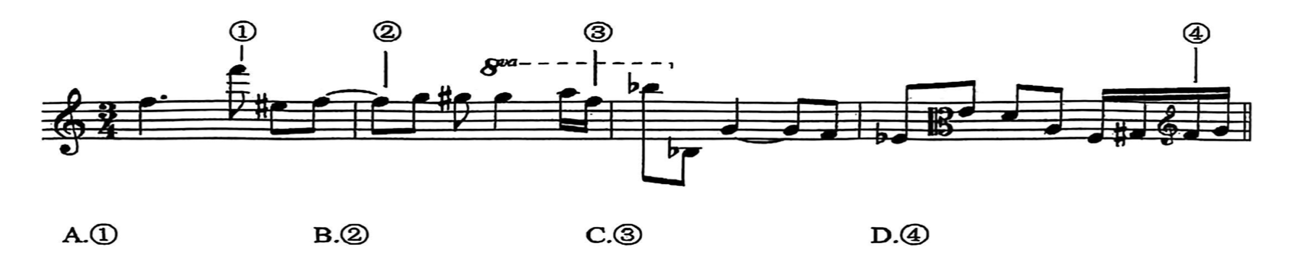


**15.Among the following sets of notes, which one contains chromatic semitones ( ).**


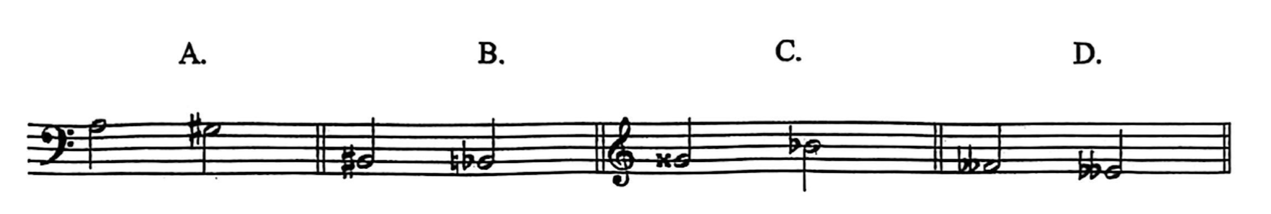


**16．1. The correct statement about pitch is ( )**

- 1. A. Pitch is determined by the duration of the vibrating body
  2. B. Pitch is determined by the frequency of vibration
  3. C. Pitch is determined by the amplitude
  4. D. Pitch is determined by the different component relationships of the vibration

**17.** **The key signature that should be used for the following melody fragments is ( ).**


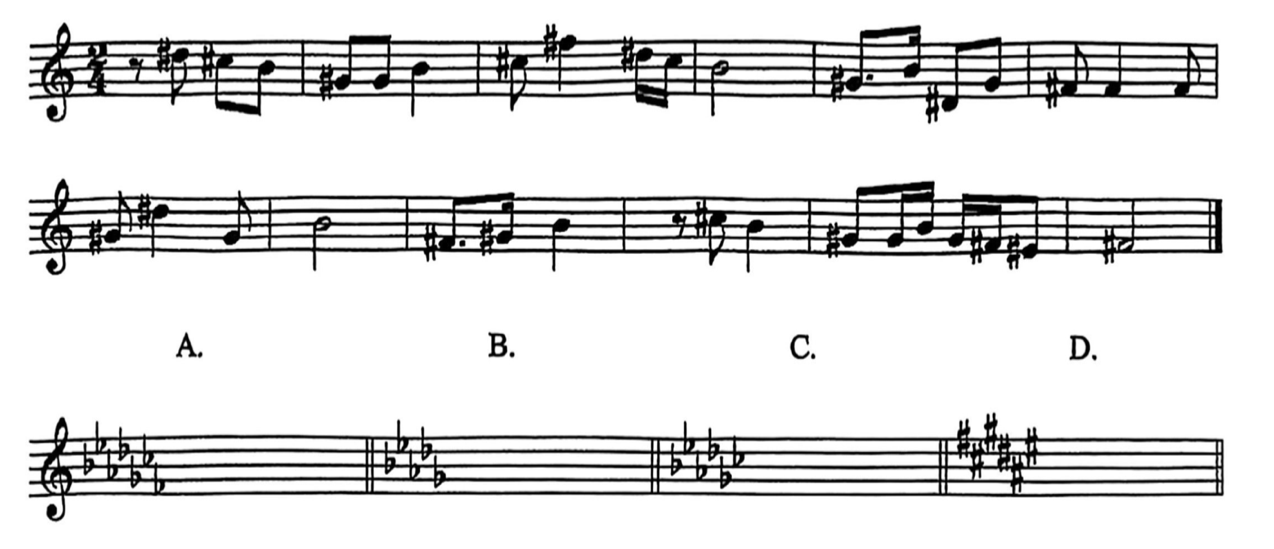


**18.Which interval is considered the most stable in a melody?**

- 1. A. Perfect octave
  2. B. Minor third
  3. C. Augmented fourth
  4. D. Major seventh

**19.What is the relationship between the notes in a four-bar melody using perfect fourth intervals?**

- 1. A. Each pair of notes is separated by a fourth
  2. B. Each pair of notes is separated by a fifth
  3. C. Each pair of notes is separated by a third
  4. D. Each pair of notes is separated by a seventh

**20. Select the equivalent sounds for the following sounds ( ) .**

**
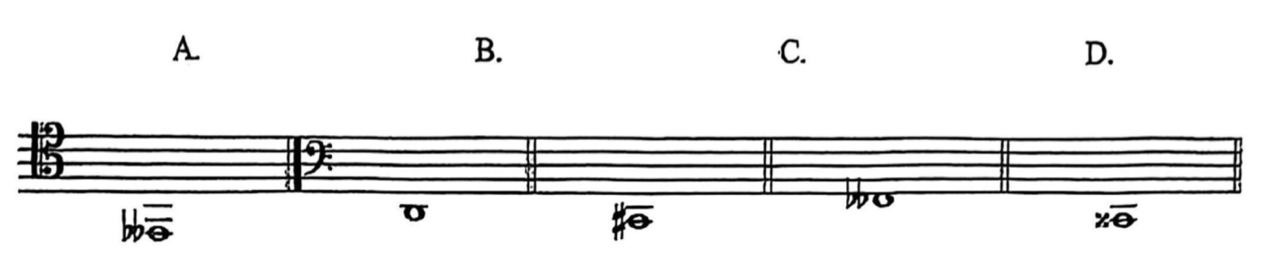
**

**21. Select the correct ellipsis symbol for the following fragments ( ).**

**
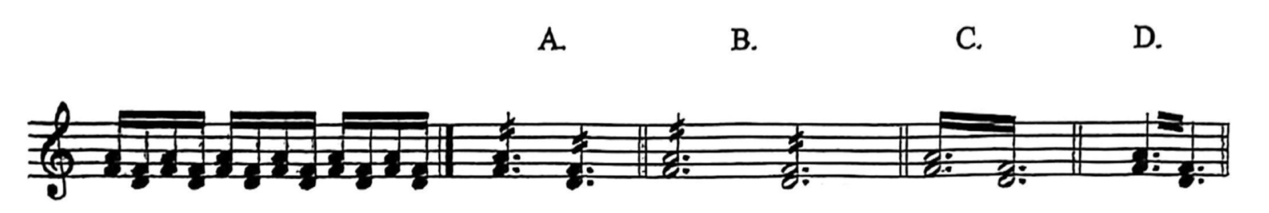
**

**22.Select the correct ellipsis symbol for the following fragments ( ).**

**
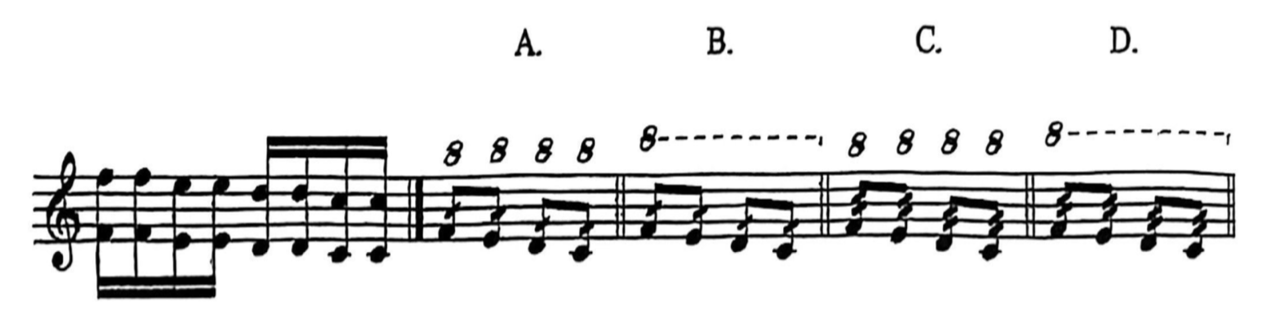
**

**23.Write the actual performance of the following ornamental notes ( ).**

**
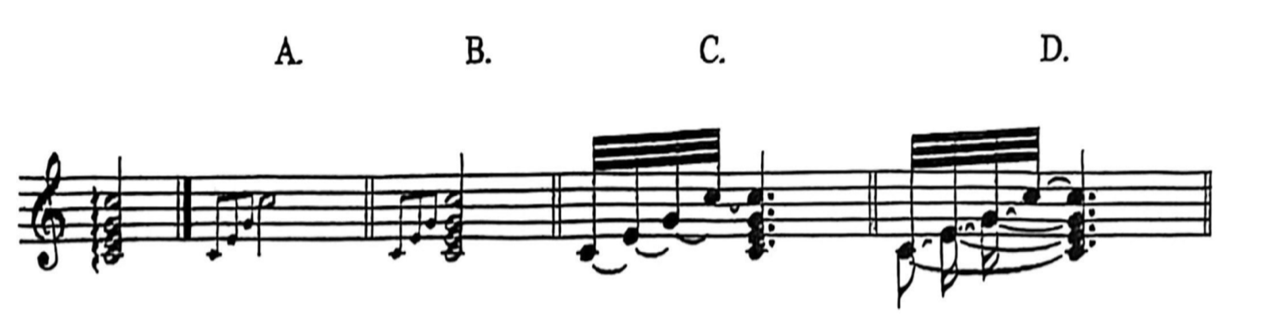
**

**24. Select the correct performance order ( ).**

**
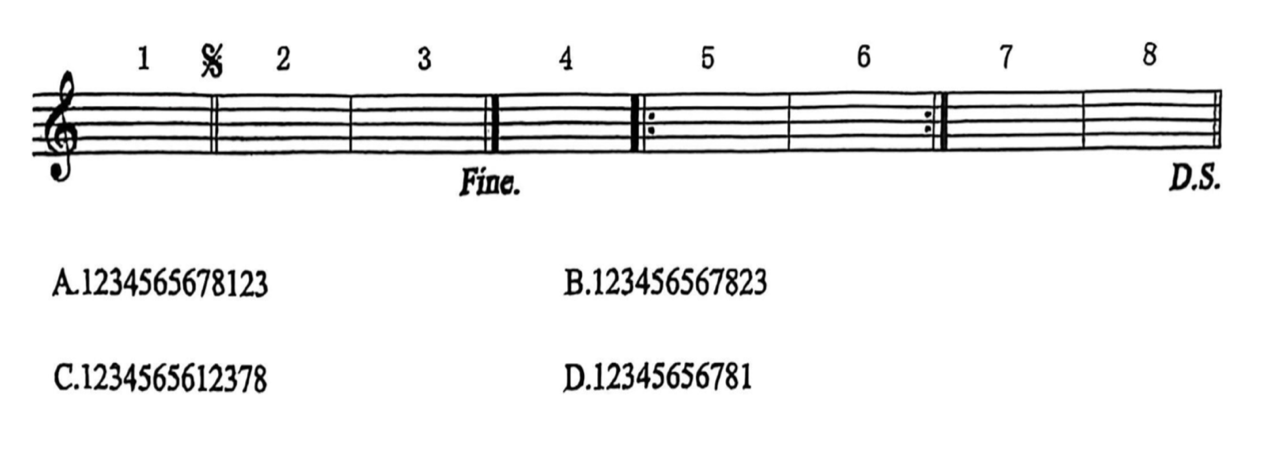
**

**25.The following examples are selected from Bach's “Musical Offering.” In these four excerpts, which option has the correct chromatic scale ( ).**

**
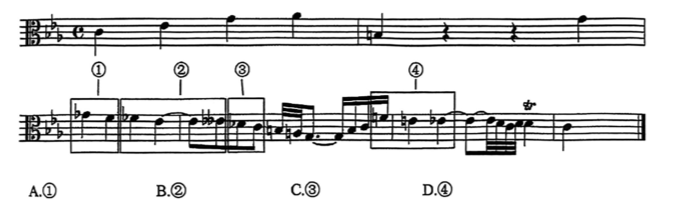
**

**B: Music Element Skills (Pre-test)**

**Name: _________________________ Date: _________________________**

**1.Change the following minor intervals to major intervals using accidentals and write the names of the intervals before and after the change. ( 5 )**

**
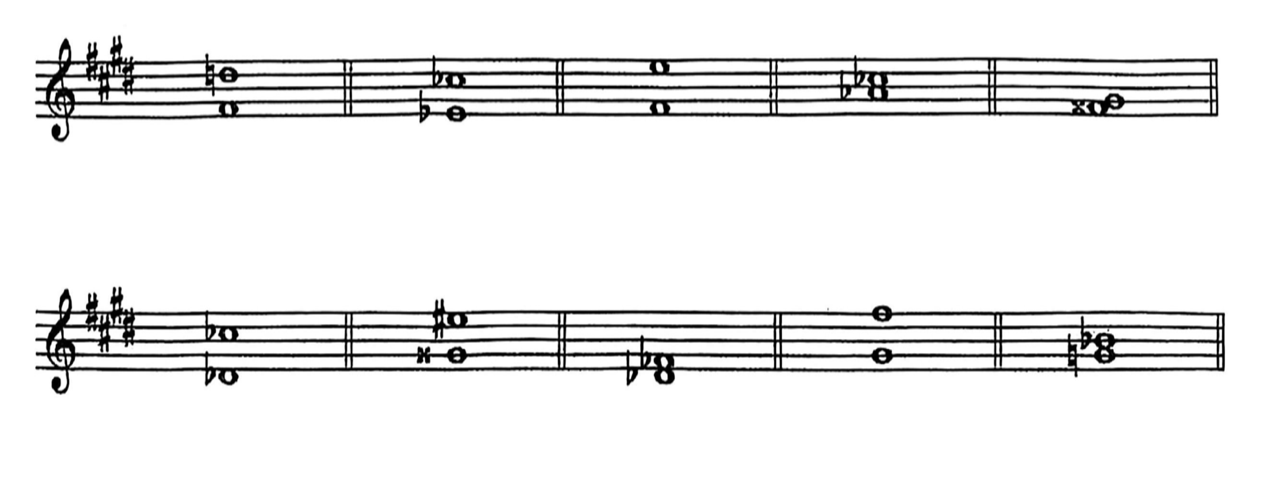
**

**2.** **Use a single note to replace the total duration of the following slurred notes. (2)**

**
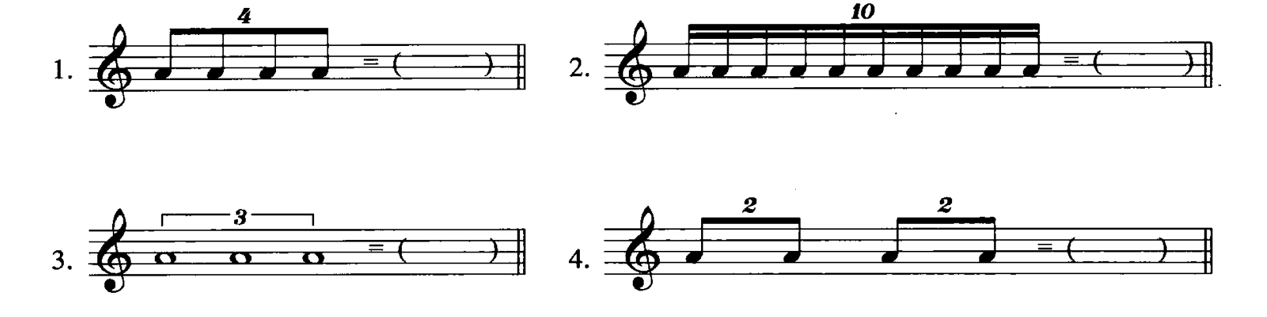
**

**3.Correct the errors in the following notation. (4 )**

**
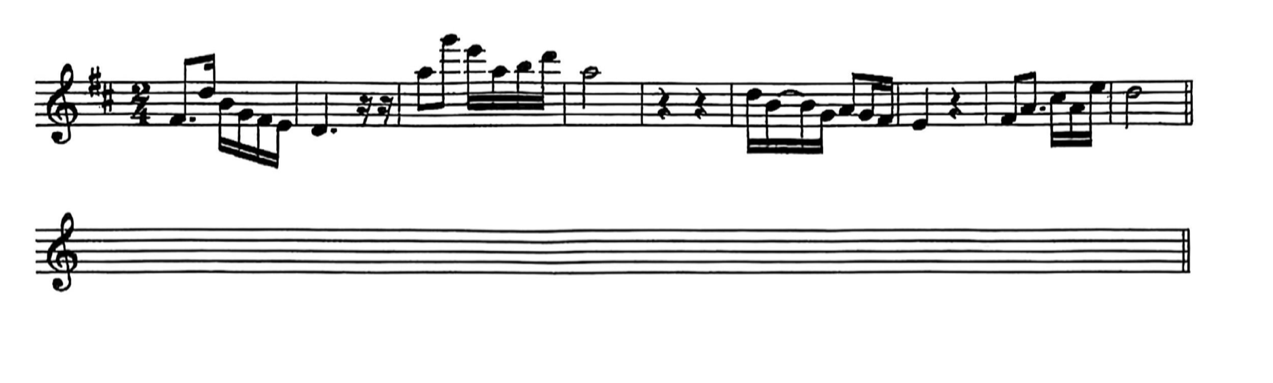
**

**4.Move the following melody to the same pitch. (4)**

**
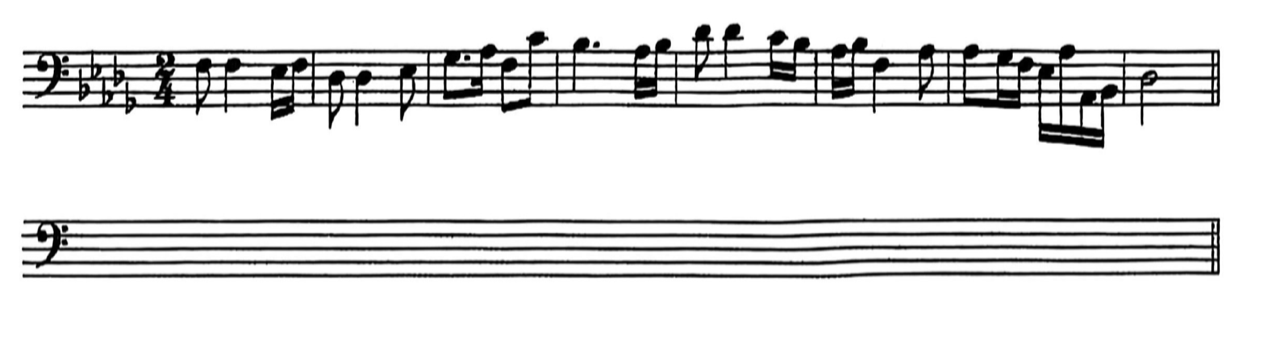
**

**5.Using d2 as the middle tone, write the scales of the natural major, harmonic major, natural minor, and melodic minor keys. ( 4 )**

**
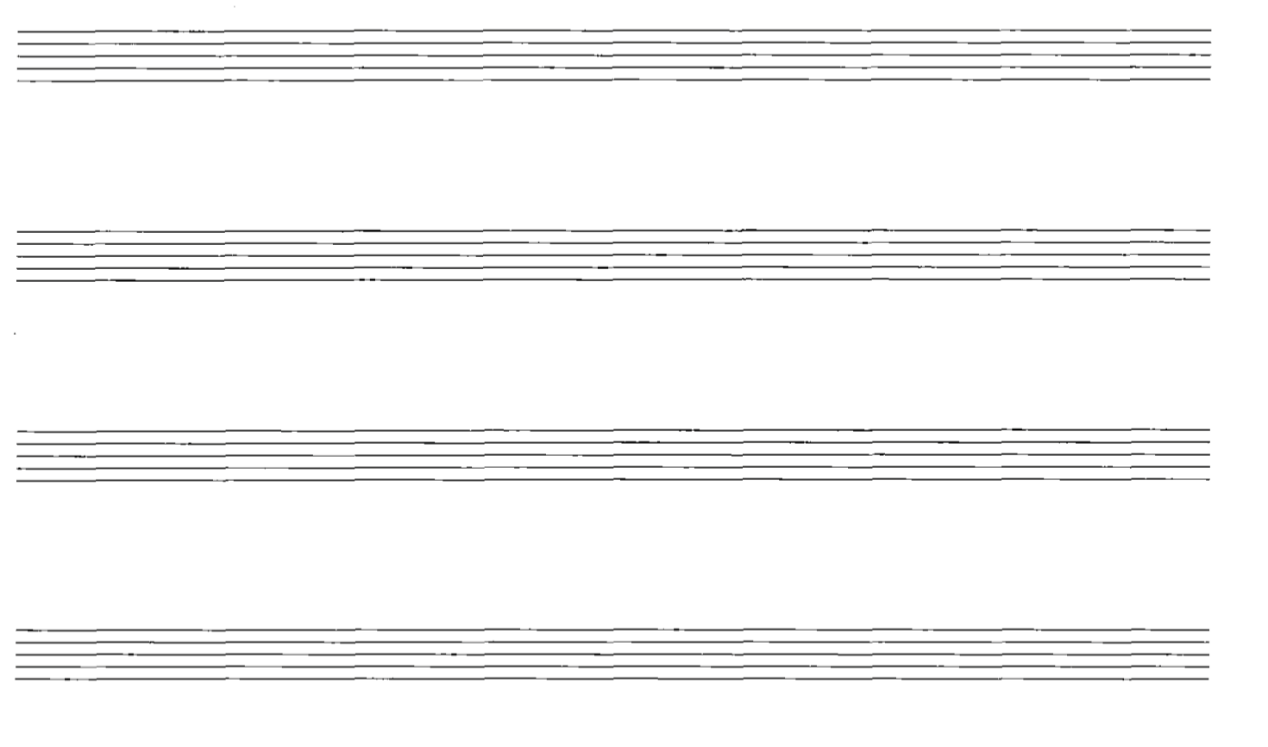
**

**6.Write down the actual performance effects of the following examples. (2 )**

**
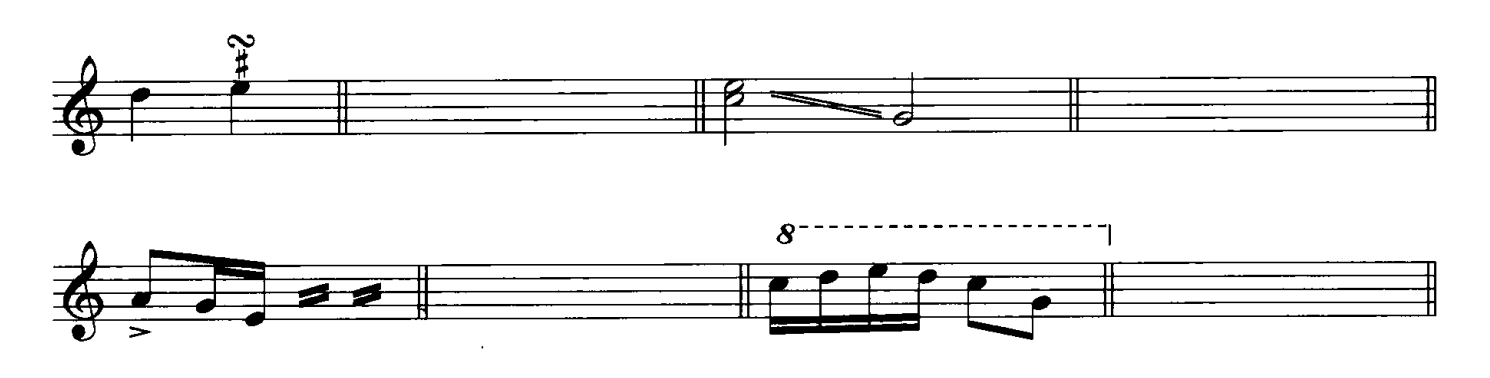
**

**7.Divide the following melody into measures according to the specified time signature and combine them correctly according to the note value grouping method. ( 4 )**

**
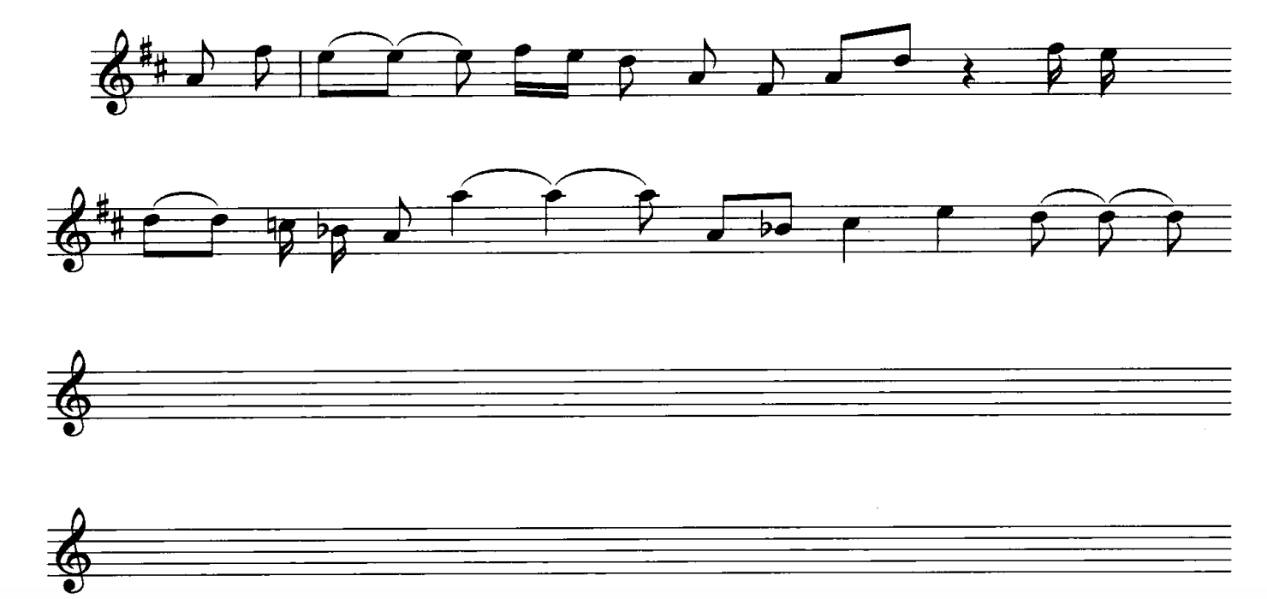
**

**A: Test Paper: Student Cognitive Abilities (Post-test)**

**Name: ______________________ Date: _________________________**

**Instructions: Single Choice Question（25）**

**1.Which of the following four intervals is not a natural interval ( ).**


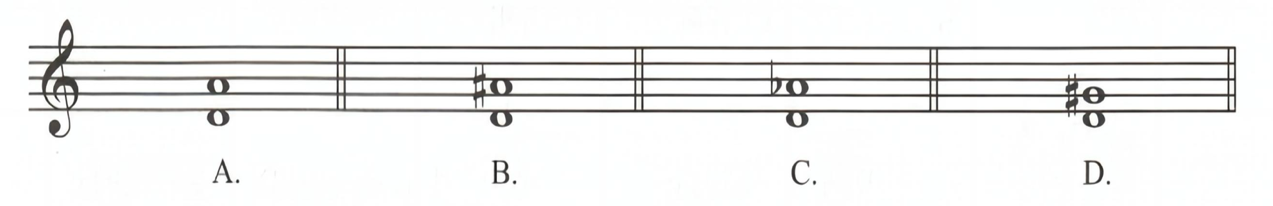


**2.Which of the following four chords is a consonant chord ( ).**


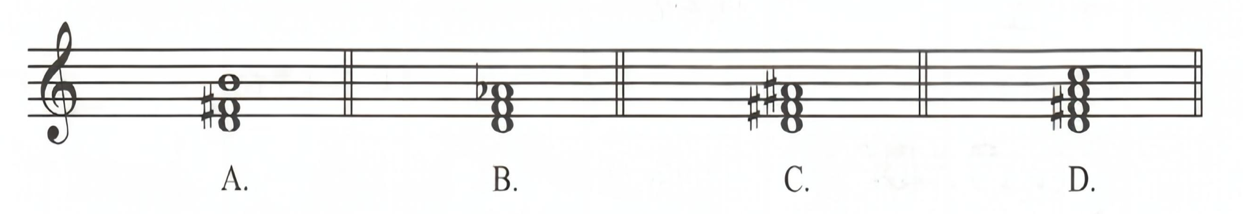


**3.** **Which of the following four chords is a half-diminished seventh chord ( ).**

**
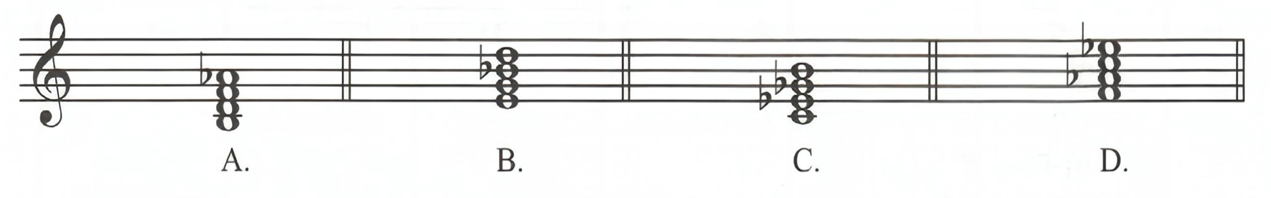
**

**4.**
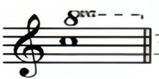
 **Which group do the notes in the staff belong to ( ) .**

- 1. A. Small character group c
  2. B. Small character group 1 c
  3. C. Small character group 2 c
  4. D. Small character group 3 c

**5.
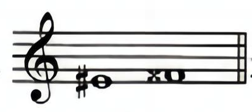
** **The relationship between these two sounds is ( ).**

- 1. A. Natural whole tone
  2. B. Altered whole tone
  3. C. Natural half tone
  4. D. Altered half tone

**6.
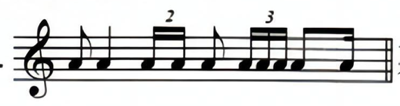
** **Its total time value is equal to ( ).**


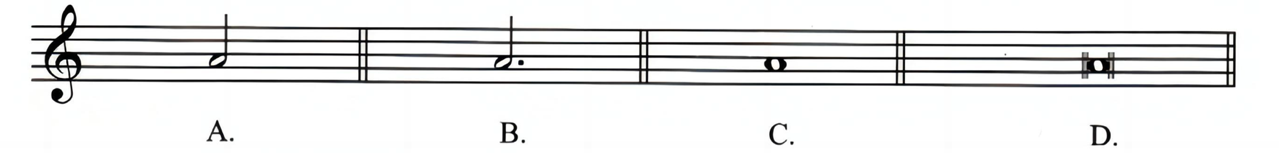


**7.Which of the following four musical terms is an expression term ( ).**

- 1. A. Lento
  2. B. ritenuto
  3. C. assai
  4. D. cantabile

**8.Arranging the tones in a musical system according to certain pitch relationships and order is called ( ).**

- 1. A. Pitch
  2. B. Tone row
  3. C. Scale
  4. D. Tone group

**9.The interval with a number of tones equal to 3 is ( ).**

- 1. A. Double fourth
  2. B. diminished fifth
  3. C. perfect fifth
  4. D. diminished sixth

**10.The following examples are selected from Bach's “Musical Offering.” In these four excerpts, which option has the correct chromatic scale ( ).**

**
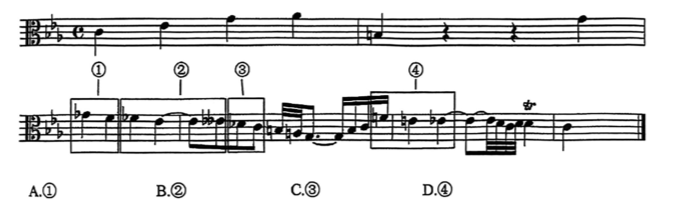
**

**11.The transposition of a major sixth interval is ( ).**

- 1. A. Altered interval
  2. B. Perfect interval
  3. C. Imperfect interval
  4. D. Dissonant interval

**12.** **The key signature that should be used for the following melody fragments is ( ).**


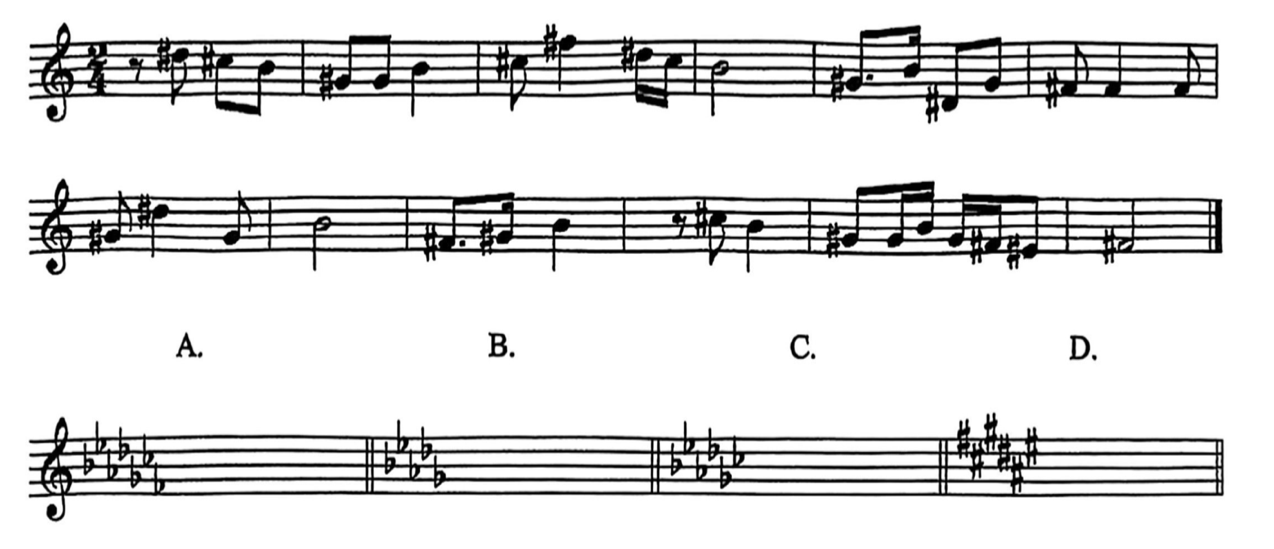


**13.Which interval is considered the most stable in a melody?**

- 1. A. Perfect octave
  2. B. Minor third
  3. C. Augmented fourth
  4. D. Major seventh

**14.The following are not
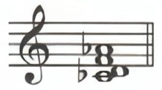
 equal chords ( ).**


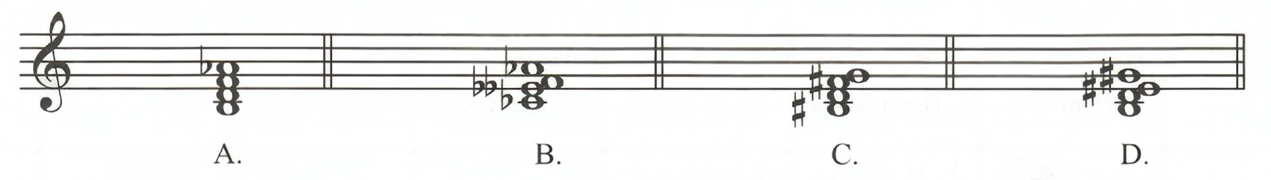


**15.In all modes, the augmented second interval exists in ( ).**

- 1. A. Natural major and minor scales
  2. B. Harmonic major and minor scales
  3. C. Melodic major and minor scales
  4. D. Seven-tone classical music

**16.Which of the following chords does not belong to the major triad of D major ( ).**
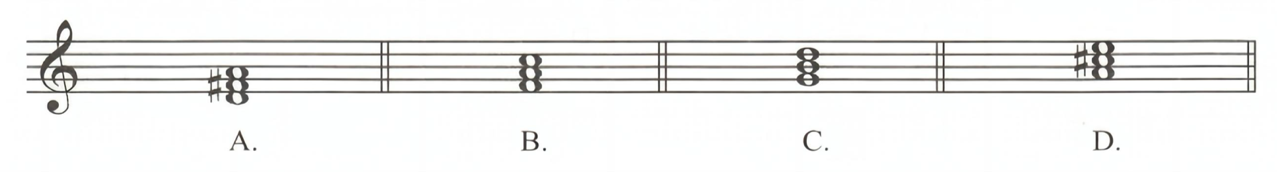


**17.See the following examples. In numbers ① to ④, which ones are in group f3( ) .**


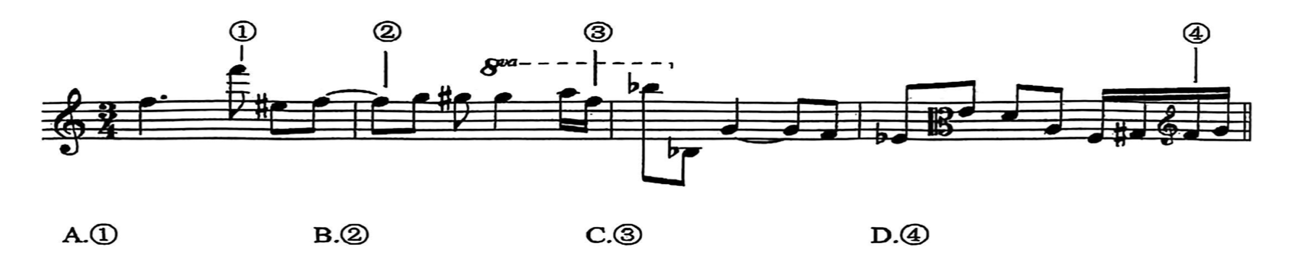


**18.Among the following sets of notes, which one contains chromatic semitones ( ).**


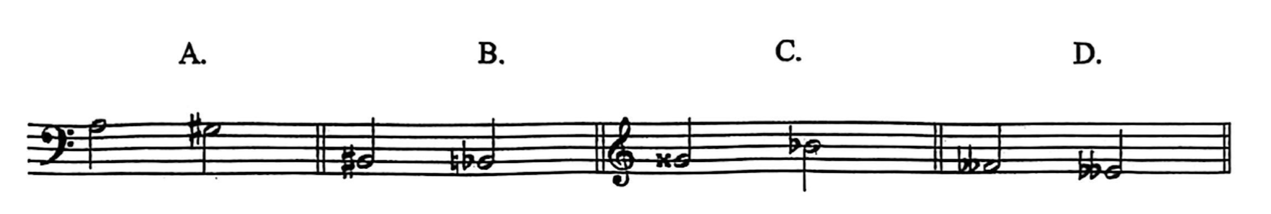


**19.What is the relationship between the notes in a four-bar melody using perfect fourth intervals?**

- 1. A. Each pair of notes is separated by a fourth
  2. B. Each pair of notes is separated by a fifth
  3. C. Each pair of notes is separated by a third
  4. D. Each pair of notes is separated by a seventh

**20. Select the equivalent sounds for the following sounds ( ) .**

**
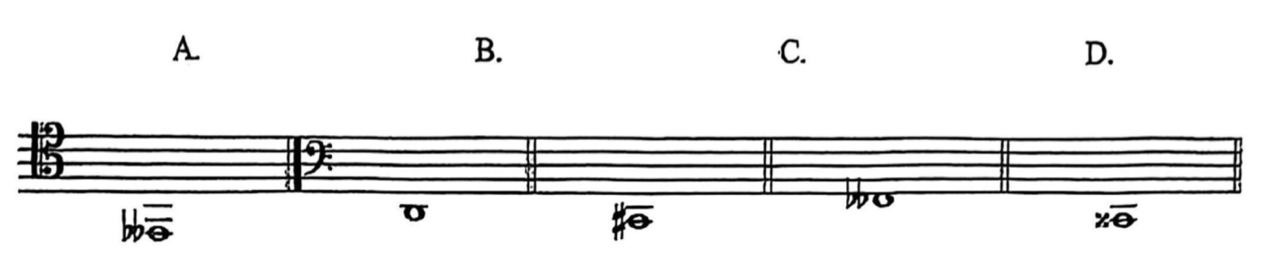
**

**21. Select the correct ellipsis symbol for the following fragments ( ).**

**
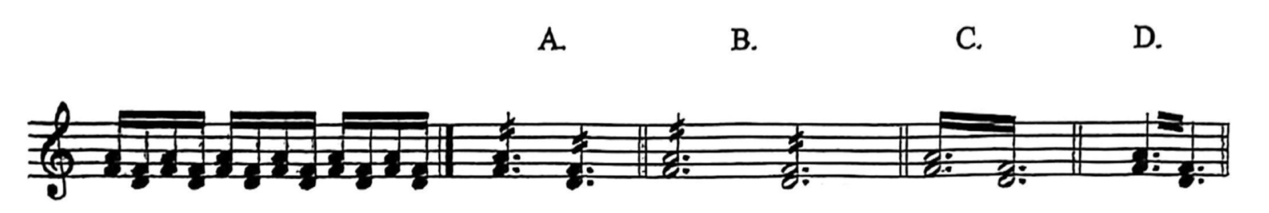
**

**22.Select the correct ellipsis symbol for the following fragments ( ).**

**
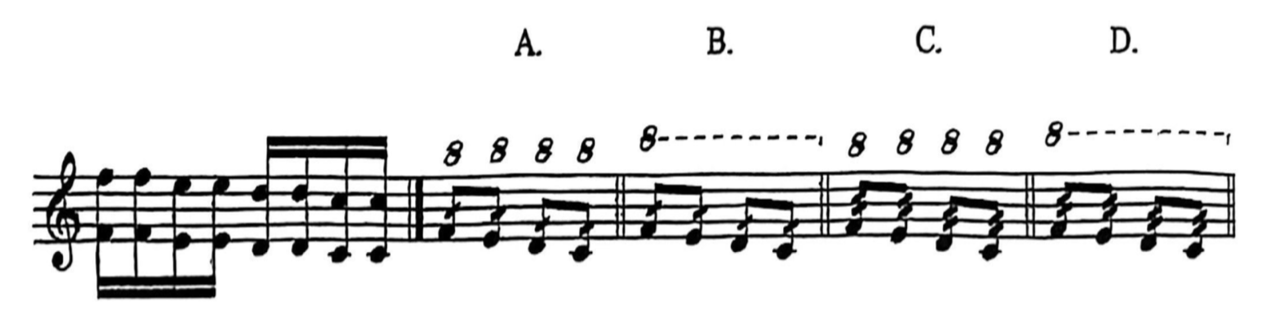
**

**23.Write the actual performance of the following ornamental notes ( ).**

**
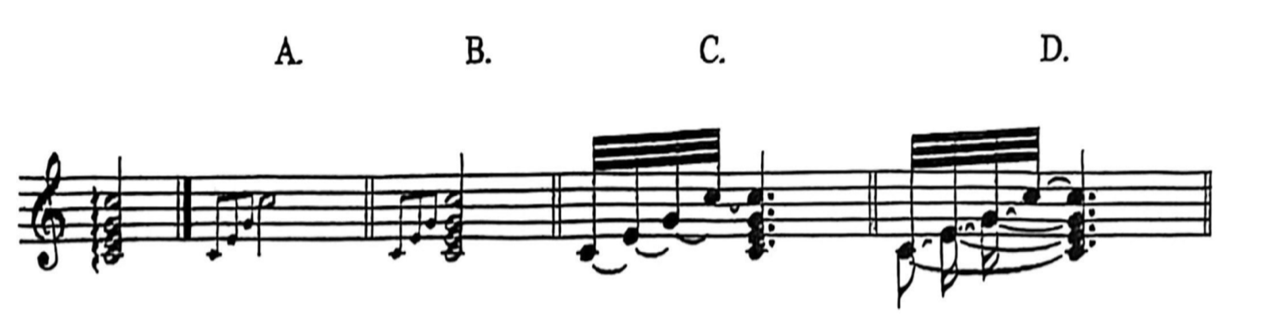
**

**24. Select the correct performance order ( ).**

**
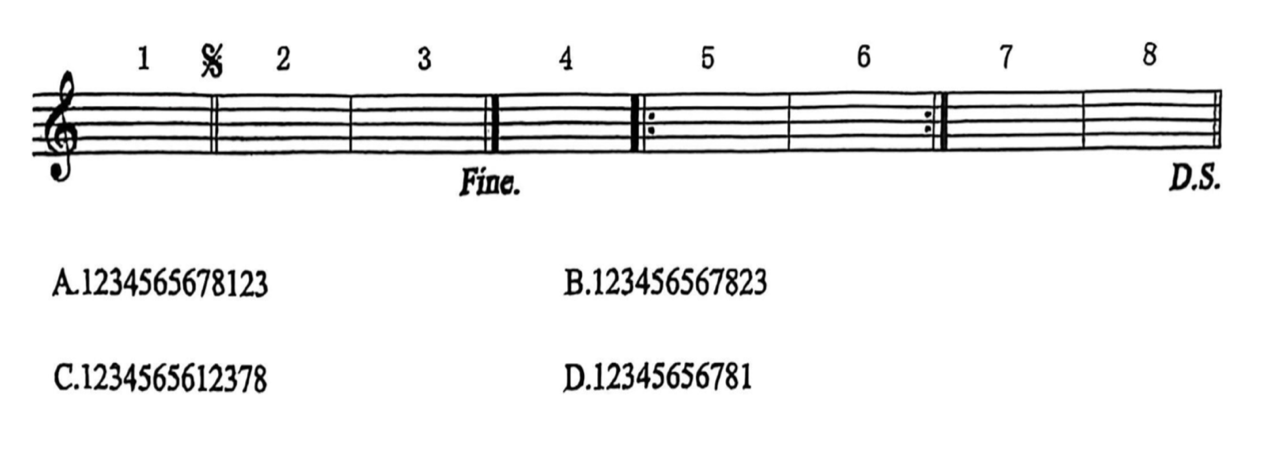
**

**25．1. The correct statement about pitch is ( )**

- 1. A. Pitch is determined by the duration of the vibrating body
  2. B. Pitch is determined by the frequency of vibration
  3. C. Pitch is determined by the amplitude
  4. D. Pitch is determined by the different component relationships of the vibration

**B: Music Element Skills (Post-test)**

**Name: _________________________ Date: _________________________**

**1.Move the following melody to the same pitch. (4)**


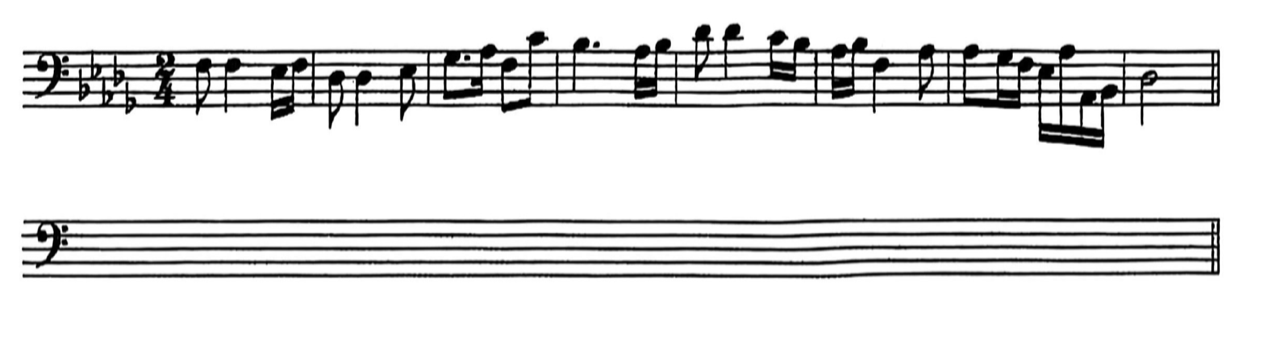


**2. Divide the following melody into measures according to the specified time signature and combine them correctly according to the note value grouping method. (4)**


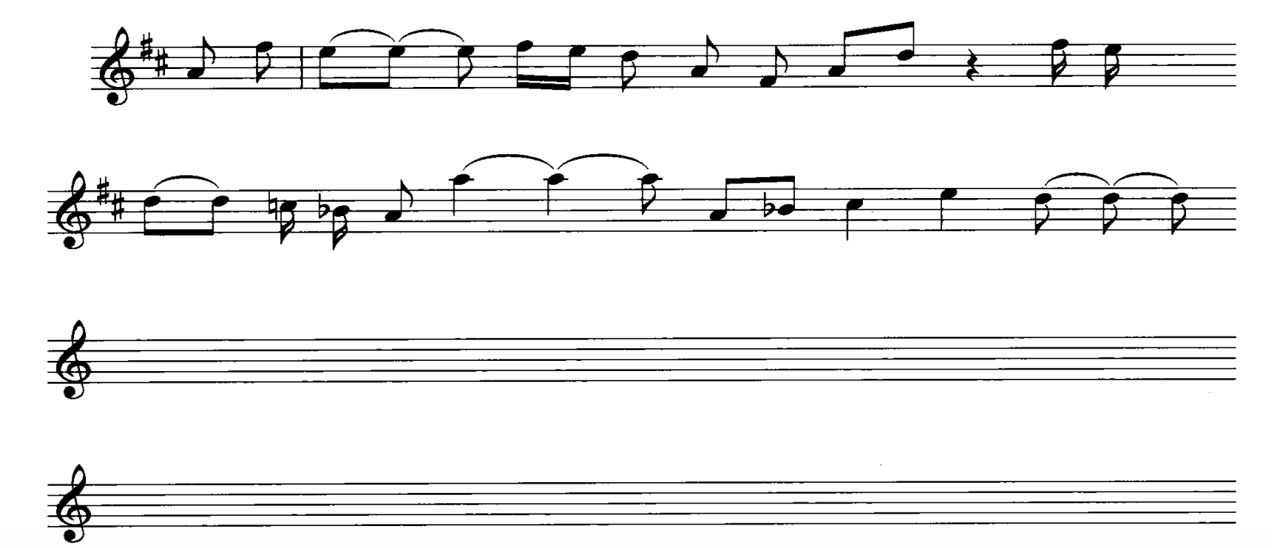


**3.Change the following minor intervals to major intervals using accidentals and write the names of the intervals before and after the change. (5)**


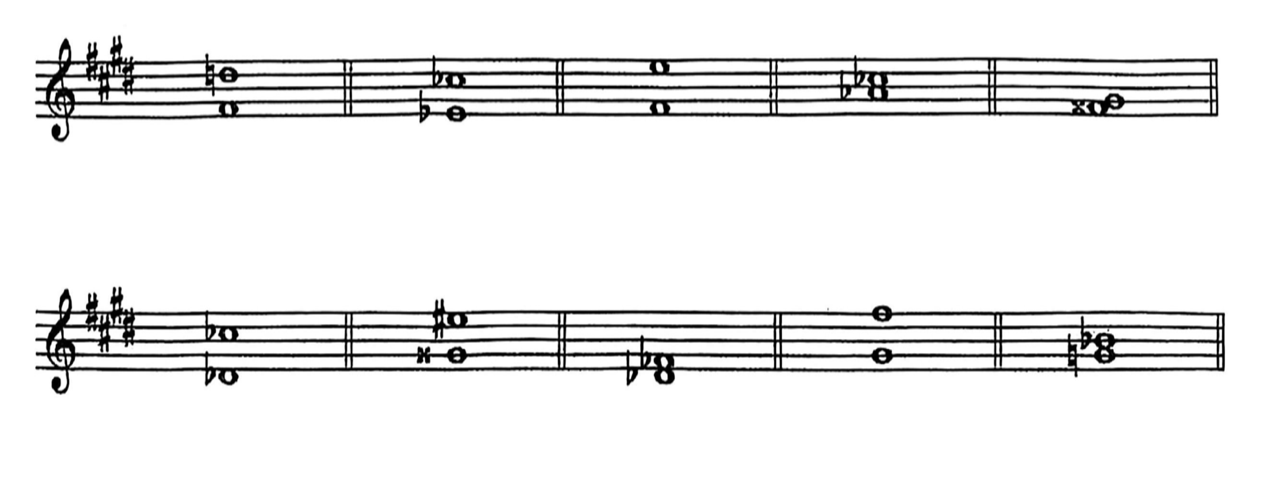


**4. Use a single note to replace the total duration of the following slurred notes. (2)**


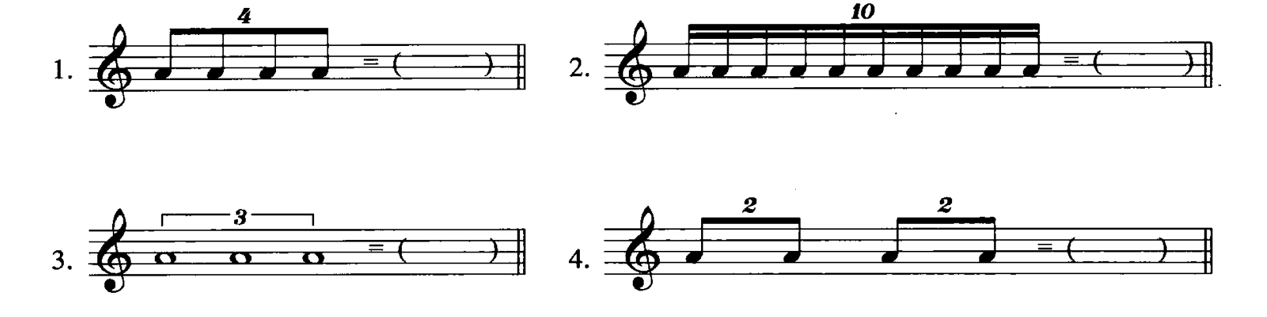


**5.Correct the errors in the following notation. （4）**


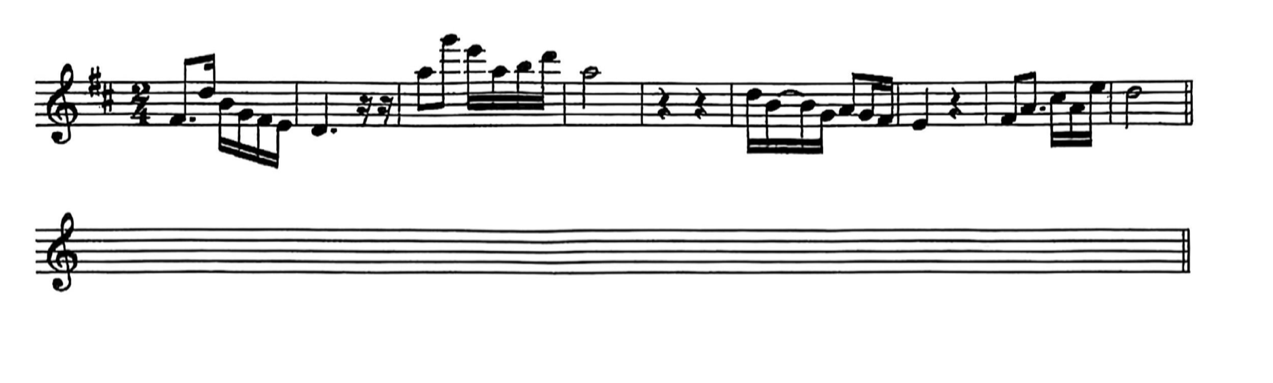


**6.Using d2 as the middle tone, write the scales of the natural major, harmonic major, natural minor, and melodic minor keys. (4)**


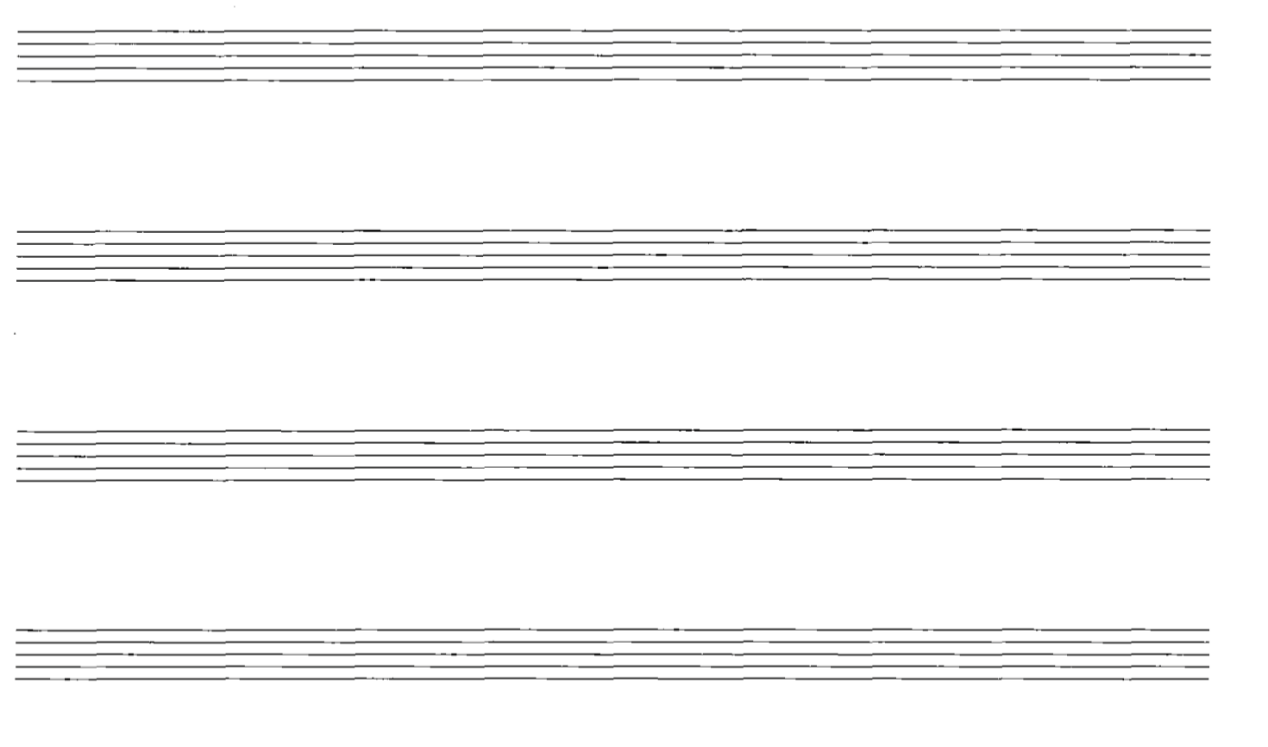


**7.Write down the actual performance effects of the following examples. (2)**


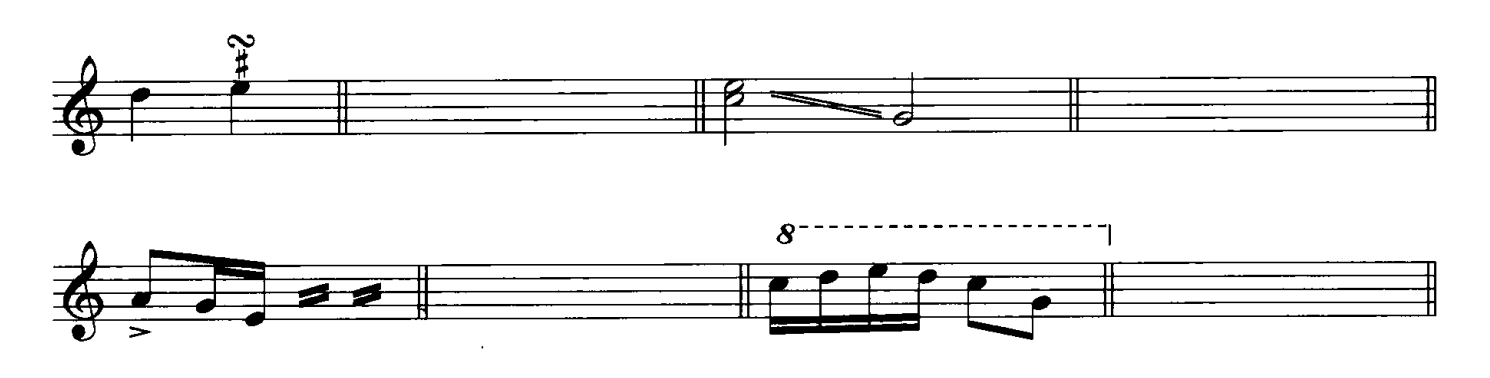

Supplement: S1 File — (DOCX) [file pone.0337590.s001.docx]
