# Supplementary material for: Development of flipped classroom module fcm for music theory instruction: An innovative approach to music education
Source: PLoS One. 2025 Nov 21;20(11):e0337590. doi: 10.1371/journal.pone.0337590 (PMC12637912; doi:10.1371/journal.pone.0337590)
Supplement: Attachment — Submitted filename: [10-Oct] Response to Reviewers(1)(1).docx [file pone.0337590.s005.docx]

|  | **Manuscript ID: PONE-D-25-42105** |
| --- | --- |
|  | **RESPONSE TO EDITORS:21- October -2025** |

Response to Reviewer’s Comments

I am grateful for your insightful comments. I have been able to incorporate changes to reflect all of the suggestions provided by you before. I have also highlighted the changes in the revised manuscript with track changes for your review.

| **No** | **Addressed Points** | **Response** | **Page** |
| --- | --- | --- | --- |
| **Reviewer: 1** | | | |
| 1. | Is there anything called “Western flipped Classroom Models” if yes, justify you claim with source(s) | Thank you for your comment. We have revised the phrase to “flipped classroom models developed primarily in Western educational contexts” for clarity and added supporting sources to justify this claim. | Page 2, chapter 1.1, paragraph 3. |
| 2. | What’s the full meaning of LMS? Before using abbreviation there is a need to state the full meaning at first use. Then subsequently the abbreviation can be used. | Thank you for your comment. We have revised the manuscript to spell out the full term at its first mention as “Learning Management System (LMS)” and used the abbreviation in subsequent occurrences for clarity and consistency. | Page 5, chapter 2.3, paragraph 3. |
| 3. | Undergraduates should be sufficient rather than saying “undergraduate students” | Thank you for your suggestion. The word “students” has been removed. | The whole manuscript (page 5 and 6). |
| 4. | Who is the instructor? | The instructor was a university lecturer with over ten years of experience teaching undergraduate music theory courses and was responsible for implementing the flipped classroom module. | Page 7, chapter 3.3 |
| 5. | What’s your justification for having Flipped classroom to be 30 and control group is also 30. I would have expected you to use intact class. | Thank you for your comment. In this study, two intact classes were selected from a total of five first-year music performance classes. Each class had 30 students, while the other three classes had uneven sizes, which would have limited comparability. Using these two intact classes ensured balanced group sizes, ecological validity, and natural learning conditions, with both groups taught by the same instructor under identical instructional settings except for the teaching approach. | Page 7 |
| 6. | Both groups consisted of two intact classes from the same academic year and music theory program to ensure ecological validity and minimize cross-group contamination. | Thank you for the comment. The text has been revised to clarify that two intact classes (30 students each) were selected from five available classes to ensure equal group sizes and ecological validity. Both classes were taught by the same instructor under identical conditions, differing only in instructional approach. | Page 7 |
| 7. | There is a need to state your formulated hypotheses after Literature Review. | Thank you for your comments, I added a new chapter 3.1 research hypotheses to state those four hypotheses after literature review. | Page 5-6, chapter 3.1 |
| 8. | Conclusion and Recommendations | Thank you for your valuable comment. The Conclusion and Recommendations section has been revised to provide a clear summary of the study’s key findings, theoretical grounding, and practical implications. The revised section now highlights how the Flipped Classroom Module (FCM) enhanced students’ cognitive understanding and practical musicianship, outlines its theoretical foundations, and offers actionable recommendations for educators and future research. This addition ensures that the section fully meets the reviewer’s expectations for clarity, coherence, and academic contribution. | P=20, chapter 5.5 |
